# Correction: Li et al. Synthesis of Bimetallic FeCu-MOF and Its Performance as Catalyst of Peroxymonosulfate for Degradation of Methylene Blue. *Materials* 2022, *15*, 7252

**DOI:** 10.3390/ma19142969

**Published:** 2026-07-10

**Authors:** Huanxuan Li, Chen Xu, Ning Li, Tao Rao, Zhong Zhou, Qingwei Zhou, Chunhui Wang, Shaodan Xu, Junhong Tang

**Affiliations:** College Materials & Environmental Engineering, Hangzhou Dianzi University, Hangzhou 310018, China; 21201129@hdu.edu.cn (C.X.); 21201215@hdu.edu.cn (N.L.); raotao990519@163.com (T.R.); 222200110@hdu.edu.cn (Z.Z.); zhouqw@hdu.edu.cn (Q.Z.); wch@hdu.edu.cn (C.W.); xusd@hdu.edu.cn (S.X.)

There were errors in the original publication [1].

In the Abstract, “CuO_2_” has been updated to “Cu_2_O”.

In Section 2.4, “Analytical Methods”, “scanning electron microscope (ZEISS Ultra 55, Jena, Germany)” has been updated to “scanning electron microscope (FEI Apreo S, Waltham, MA, USA)”, and “transmission electron microscope (FEI Tecnai F30, Hillsboro, OR, USA)” has been updated to “transmission electron microscope (JEOL JEM 2100F, Tokyo, Japan)”.

The authors state that the scientific conclusions are unaffected. This correction was approved by the Academic Editor. The original publication has also been updated.
